# Genotype-phenotype correlation of neurodevelopmental disorders in patients with dystrophinopathies

**DOI:** 10.1016/j.jped.2025.01.014

**Published:** 2025-06-06

**Authors:** Fabrício M. Soares, Bruna F. Rosa, Gabriela M. Giordani, Daniele L. Rocha, Ana Carolina Brusius-Facchin, Michele M. Becker, Jonas Alex M. Saute

**Affiliations:** a Universidade Federal do Rio Grande do Sul, Programa de Pós-Graduação em Medicina: Ciências Médicas, Porto Alegre, RS, Brazil; b Hospital de Clínicas de Porto Alegre, Serviço de Genética Médica, Porto Alegre, RS, Brazil; c Hospital de Clínicas de Porto Alegre, Unidade de Neurologia Infantil, Porto Alegre, RS, Brazil; d Hospital de Clínicas de Porto Alegre, Serviço de Neurologia, Porto Alegre, RS, Brazil; e Universidade Federal do Rio Grande do Sul, Departamento de Medicina Interna, Porto Alegre, RS, Brazil

**Keywords:** Genotype-phenotype correlation, Dystrophin isoforms, Dystrophinopathies, Autism spectrum disorder, Attention-deficit/hyperactivity disorder, Obsessive-compulsive disorder

## Abstract

**Objective:**

Neurodevelopmental disorders are frequently and heterogeneously diagnosed among patients with dystrophinopathies. The authors aimed to evaluate how the symptoms of Attention-Deficit/Hyperactivity Disorder (ADHD), Obsessive-Compulsive Disorder (OCD), or Autism Spectrum Disorder (ASD), and genotype are related to *DMD* genotype.

**Methods:**

In an observational cross-sectional study, standardized instruments were applied to 50 participants and their caregivers, mainly from a reference center for rare diseases in Southern Brazil (*n* = 38) or other Brazilian centers (*n* = 12). Participants were divided according to genotype and affected dystrophin isoforms.

**Results:**

The overall diagnostic rate of symptoms of ASD was 34 %, similar to OCD (35.5 %), with half of the participants (51.4 %) having symptoms compatible with ADHD. Cerebral isoforms were affected in more than half of the participants (52 %). Symptoms compatible with ASD and OCD, and Childhood Autism Rating Scale (CARS) scores were associated with genotype and impairment of cerebral isoforms of dystrophin.

**Conclusions:**

The prevalence of symptoms compatible with ASD (and higher CARS scores) and OCD among patients with dystrophinopathies are related to the position of the causal variant in *DMD* and the consequent involvement of cerebral isoforms, indicating an important genotype-phenotype correlation. The diagnosis of a patient with a genotype that affects these isoforms indicates the need for neuropsychological assessment and multidisciplinary follow-up.

## Introduction

Dystrophinopathies are neuromuscular disorders caused by the absence/reduction of dystrophin function, a product of the *DMD* gene located on Xp21. Single nucleotide or copy number variations of *DMD* result in clinical forms whose severity is related to the residual protein activity [1,2].

At the most severe end of the dystrophinopathy spectrum is Duchenne muscular dystrophy (DMD), which occurs when there is a complete absence of dystrophin. DMD affects 1 in 5,000 live-born boys [3]. It is characterized by delayed motor milestones, pseudo-hypertrophy of calf muscles, and progressive muscle weakness that invariably leads to loss of ambulation in early puberty and cardiopulmonary complications in the third decade of life [4,5]. On the other hand, Becker muscular dystrophy (BMD) is marked at the cellular level by residual dystrophin activity and is clinically manifested by muscle cramps and weakness. BMD is also noteworthy for its cardiac muscle involvement [4].

Despite their muscular phenotype, neurodevelopmental disorders have been observed since the description of DMD and can be the initial presentation of the disease [6,7]. In a recent systematic review involving 3121 participants, the prevalence of neurodevelopmental disorders such as autism Spectrum Disorder (ASD), obsessive-compulsive disorder (OCD), and attention-deficit/hyperactivity disorder (ADHD) was higher than expected for the general population, reaching rates as high as 21 %, 33 %, and 50 %, respectively [8].

Initially associated with functional impairments related to the disease or even environmental factors, the hypothesis of a biological role of dystrophin in central nervous system development was raised from the observation of the concordance of intelligence quotient between affected siblings with dystrophinopathies [9]. Subsequently, this role was found to be related to the neuronal expression of dystrophin during neurodevelopment [10].

**Figure 1:**
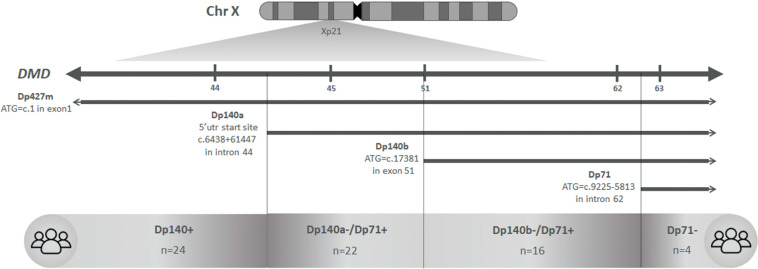
Schematic representation of chromosome X, DMD, isoforms of interests and studied groups. Exons close to each isoform’s start codons are indicated on top for reference. In the middle, chromosomal position is given for studied isoforms. Below, n represents the number of participants in each studied group. Analysis considering the group Dp140b-/Dp71+ had a total of 44 participants, once 6 participants had mutations between exons 44 and 51.

The DMD gene consists of 79 exons and 7 promoters, generating tissue-specific isoforms. The largest isoform, Dp427, with 427 kDa, is expressed in skeletal and cardiac muscle. Smaller isoforms like Dp140 and Dp71 are abundant in the brain, and their promoters are located in the second half of the DMD gene. Variants starting from exon 51 affect Dp140 in addition to muscle isoforms, and variants starting from exon 63 affect all dystrophin isoforms, including Dp71 (Figure 1) [11].

The association between the manifestation of neurodevelopmental disorders and the genotype of DMD patients is consistent for intelligence quotient, where they perform 1.0–1.5 standard deviations below the population average, being worse among those with impairment of brain isoforms [12,13]. For other neurodevelopmental disorders, despite their increased prevalence in this group, this relationship is not yet clear [14].

In this multicenter study, the authors aimed to assess the prevalence of neurodevelopmental disorders among patients with DMD and BMD, including ASD, ADHD and OCD. Standardized and validated instruments were used to identify how these disorders were related to the participants' genotypes.

## Methods

### Design and participants

An observational cross-sectional study was conducted, in which all patients diagnosed with DMD or BMD seen at the Medical Genetics Service of the Hospital de Clínicas de Porto Alegre (HCPA) were invited to participate. Participants from other centers in Brazil were invited through a letter to the Aliança Distrofia Brasil (ADB) and through internal social media groups of the organization (for the recruitment flowchart, see Supplementary Fig. 1). ADB is considered the largest Brazilian organization of people living with muscular dystrophies, with 16 regional associations from the five regions of the country. Inclusion criteria were: (1) having a confirmed molecular diagnosis of DMD/BMD; (2) age of 4 years and older, with no upper age limit; (3) providing consent to participate in the study. Those with variants of uncertain significance in the *DMD* gene, participants with DMD caused by Xp21 contiguous gene deletion syndrome, or a diagnosis of other neurological or systemic conditions causing additional cognitive impairments, such as perinatal asphyxia, stroke, or trauma, were excluded.

### Isoforms of interest

For the comparative analysis of the frequencies of disorders studied, participants were divided into groups based on genotype and its affected isoforms. Subgroups were defined as Dp140+, Dp140a-/Dp71+, Dp140b-/Dp71+, and Dp71-. Dp140 has its promoter in intron 44 and the start codon in exon 51. For comparative purposes, participants with variants located beyond exon 44 were defined as Dp140a- and beyond exon 50 as Dp140b [13].

### Diagnostic instruments for neurodevelopmental disorders

Participants were evaluated for ASD using the Childhood Autism Rating Scale (CARS), which consists of 15 subitems that assess behaviors known to be impaired in ASD with a total score ranging from 15 to 60 [15]. ADHD was assessed using the SNAP-IV, an instrument that uses the ADHD symptoms listed in the Diagnostic and Statistical Manual of Mental Disorders (DSM-IV). It consists of 18 items divided into subareas (hyperactivity and inattention), which can be scored from "not at all" to "very much," based on the frequency of symptom manifestations [16]. The presence of symptoms of OCD was assessed using the Yale-Brown Obsessive Compulsive Scale for Children (CY-BOCS), which consists of ten questions that assess the time spent, interference caused, related distress, resistance, and degree of control over obsessive and compulsive thoughts and behaviors in children and adolescents, resulting in a score ranging from 0 to 40 points [17].

Participants with a score of 30 or higher on CARS, with at least six "quite a bit" or "very much" responses on the SNAP-IV, or a score of 16 or higher on the CY-BOCS, were considered affected by one or more of the assessed disorders [15, 16, 17]. As the project began during periods of widespread social isolation resulting from the COVID-19 pandemic; to minimize physical contact and gatherings, diagnostic instruments were administered through online video interviews.

### Statistical analysis

Normal distribution was assessed using the Shapiro-Wilk test and histograms. Data were presented as frequencies and percentages, mean and standard deviation, or median and interquartile range. Scores from the CARS and CY-BOCS scales were analyzed as continuous and categorical variables, and SNAP-IV was assessed categorically. Score comparisons between different subgroups were performed using unpaired Student's *t*-test or Mann-Whitney *U* test. Analysis of variance (ANOVA) or the Kruskal-Wallis test were used when comparing >2 groups. Differences between the frequencies of the disorders studied and genotypes were assessed by the chi-square test or Fisher's exact test, providing a 95 % confidence interval for the calculated odds ratio. The p-value of < 0.05 was considered significant. IBM SPSS Statistics 20 was used for the analysis.

### Ethical considerations

The study was approved by the Research Ethics Committee of HCPA (2019–0384), and all participants or their legal representatives provided informed consent/assent prior to participation.

## Results

Fifty-two participants and their guardians were interviewed between January 2021 and January 2023. Forty participants were recruited at HCPA, and twelve enrolled from other Brazilian centers through invitation by ADB. Two participants were excluded due to a molecular diagnosis of uncertain significance. No participants were excluded due to the presence of other confounding clinical conditions. Descriptive data regarding age, age at diagnosis, phenotype, molecular diagnosis, treatment, and affected isoforms are presented in Table 1. Forty-three participants exhibited the DMD phenotype, and seven participants presented the BMD phenotype. All participants had a molecular diagnosis at the time of the interview, and the main type of variation was exonic deletions or duplications (64 %) of the *DMD* gene. All variants affected Dp427m, while cerebral isoforms were affected in more than half of the participants (52 %).

**Table 1:** Clinicogenetic characterization of the DBMD cohort.

	Median or frequency (IQR or %)			
Age in years	12y 6 m (9–17)			
Phenotype				
DMD	43/50 (86 %)			
BMD	7/50 (14 %)			
Type of variant				
CNV	32/50 (64 %)			
SNV	18/50 (36 %)			
Ambulatory stage	22/50 (44 %)			
Treatment				
Glucocorticoid	39/50 (78 %)			
Ataluren	2/50 (4 %)			
Ventilatory support	4/50 (8 %)			
	Dp140+	Dp140a-/Dp71+	Dp140b-/Dp71+	Dp71-
Dystrophin isoform	24/50 (48 %)	22/50 (44 %)	16/44 (36.4 %)	4/50 (8 %)

Note: Demographic, genetic and clinical data of DBMD cohort. Fifty patients were included in the initial analysis, but six patients had mutations between exons 44 and 51 and were excluded when group Dp140b-/Do71+ was analyzed. BMD, Becker muscular dystrophy; CNV, copy number variation; DMD, Duchenne muscular dystrophy; SNV, single nucleotide variation.

**Figure 2:**
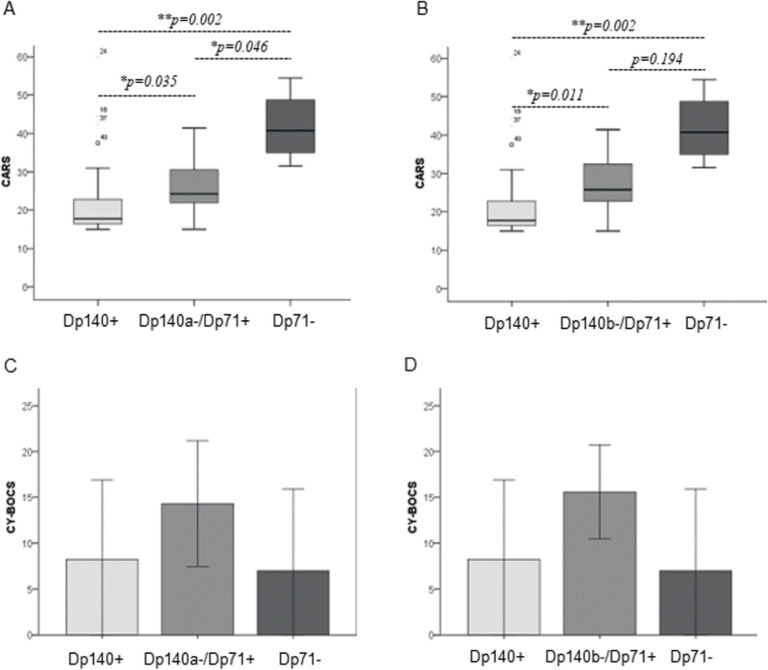
Effects of cerebral isoform impairment of dystrophin on autism and obsessive-compulsive disorder scores. CARS, childhood autism rating scale; CY-BOCS, Yale-brown obsessive compulsive scale for children. *p < 0.05; ^⁎⁎^*p* < 0.01.

All participants (*n* = 50) were assessed with the CARS, but due to specific age restrictions of the scales, 35 were evaluated with the SNAP-IV, and 31 with the CY-BOCS. The median CARS scores were 23.25 (17–31.12) in the study population, being statistically significantly higher among groups with involvement of the cerebral isoforms Dp140 and Dp71 (see Table 2), both in the analysis considering Dp140a (*p* = 0.003) and in the analysis considering Dp140b (*p* = 0.001). In the post-hoc analysis (Figure 2A), CARS scores were lower in Dp140+ participants compared to Dp140a-/Dp71+ (*p* = 0.035) and Dp71- (*p* = 0.002), with Dp140a-/Dp71+ scores being lower than those in Dp71- (*p* = 0.046), indicating a gradient where the more cerebral isoforms are affected, the higher the CARS score. When corrected for the Bonferroni test, the difference remained only between the Dp140+ and Dp71- groups (*p* = 0.005). Similarly, in the comparisons with Dp140b (Figure 2B), CARS scores were lower in Dp140+ participants compared to Dp140b-/Dp71+ (*p* = 0.011, *p* = 0.034 after Bonferroni correction) and Dp71- (*p* = 0.002, *p* = 0.007 after Bonferroni correction), with no difference in scores between Dp140b-/Dp71+ and Dp71- participants (*p* = 0.194).

**Table 2:** Characterization of non-motor features of the DBMD cohort.

	Overall	%, IQR or SD	Dp427	%, IQR or SD	Dp140a	%, IQR or SD	Dp140b	%, IQR or SD	Dp71	%, IQR or SD	*p*-value (140a)	*p*-value (140b)
CARS (score)	23.25	17–31.12	17.75	16.5–22.87	24.25	21.75–30.62	25.75	22.37–33.25	40.75	33.25–51.62	**0.003**	**0.001**
ASD	17/50	34 %	5/24	20.8 %	7/16	43.8 %	7/12	58.3 %	4/4	100 %	**0.007**	**0.001**
ADHD	18/35	51.4 %	6/14	42.9 %	9/18	50 %	5/12	41.7 %	3/3	100 %	0.196	0.166
Inattentive	17/35	48.6 %	6/14	42.9 %	8/18	44.4 %	5/12	41.7 %	3/3	100 %	0.175	0.166
Hyperactivity	10/35	28.6 %	3/14	21.4 %	6/18	33.3 %	4/12	33.3 %	1/3	33.3 %	0.747	0.773
CY-BOCS (score)	11.26	8.16	8.25	8.65	14.31	6.84	15.6	5.12	7	8.88	0.093	0.062
OCD	11/31	35.5 %	1/12	8.3 %	9/16	56.3 %	7/10	70 %	1/3	33.3 %	**0.032**	**0.011**

Note: Overall and subgroups performances on each scale and diagnostic rates by disorder. ADHD, attention-deficit/hyperactivity disorder; ASD, autism spectrum disorder; CARS, childhood autism rating scale; CY-BOCS, Yale-brown obsessive compulsive scale for children; OCD, obsessive-compulsive disorder; Statistically significant differences were highlighted in bold.

In categorical analysis, the overall diagnostic rate of symptoms of ASD was 34 %. Groups with affected cerebral isoforms had statistically significant higher prevalences (χ² = 9.674, df = 2, *p* = 0.008), with the group with more affected isoforms (Dp71-) having the highest positivity for ASD (100 %, see Table 2).

Thirty-one participants were assessed with CY-BOCS, with an average score of 11.26 (8.16), see Table 2. There were no differences in CY-BOCS scores between the isoform involvement groups when the data were analyzed continuously (Figure 2C and D), in both analyses considering isoform Dp140a-/Dp71+ (*F* = 2.593, *p* = 0.093) and Dp140b-/Dp71+ (*F* = 3.162, *p* = 0.062). However, in the categorical analysis (Table 2), the trend found in the continuous analysis was statistically significant, with a higher prevalence of symptoms of OCD in groups with affected cerebral isoforms (χ² = 6.884, df = 2, *p* = 0.032). It is worth noting that eleven participants (35.5 %) scored above 16, with only one of them in the Dp140+/Dp71+ group.

Thirty-five participants were assessed using the SNAP-IV, and over half of them (18/35, 51.4 %) presented symptoms compatible with ADHD. The combined form, with the presence of inattentive and hyperactive symptoms simultaneously, represented half of the cases (9/18). There was no association between the diagnosis of symptoms of ADHD and the impairment of cerebral isoforms (χ² = 3.260, df = 2, *p* = 0.196, comparisons with Dp140a-/Dp71+; χ² = 3.589, df = 2, *p* = 0.166, comparisons with Dp140b-/Dp71+). There were also no differences between the isoform involvement groups when analyzing the presence of hyperactivity alone (χ² = 0.583, df = 2, *p* = 0.747, comparisons with Dp140a-/Dp71+; χ² = 0.514, df = 2, *p* = 0.773, comparisons with Dp140b-/Dp71+) or inattention alone (χ² = 3.482, df = 2, *p* = 0.175, comparisons with Dp140a-/Dp71+; χ² = 3.589, df = 2, *p* = 0.166, comparisons with Dp140b-/Dp71+).

## Discussion

In this study, the authors demonstrated that the prevalence of ASD and OCD among patients with dystrophinopathies is related to the position of *DMD* variant and consequent involvement of cerebral isoforms, indicating a genotype-phenotype correlation, similar to what has been previously described for intellectual impairment. Conversely, although ADHD is present in 50 % of participants, the authors did not find an association with the involvement of cerebral isoforms.

Since the description of DMD in 1868, the common occurrence of intellectual and speech development delays among patients has been noted. Duchenne de Boulogne described the intellect of the first patients as "monotone" and their speech as difficult, initiating studies on the brain-muscle connection. Through gene expression data in brain tissue from DMD patients, it was possible to establish the temporal-spatial location of different dystrophins and gain insights into how a protein considered strictly muscular could have a role in neurodevelopment [11]. Cerebral isoforms, mainly Dp140 and Dp71, are expressed heterogeneously in the brain, in specific subcellular locations, and at specific embryonic stages, performing various cellular functions, such as membrane stabilization, cell division, intercellular adhesion, and synaptic organization. The expression of Dp140 in oligodendrocytes seems to be crucial for the myelination process, reinforcing the role of dystrophin in neurodevelopment [18].

The overall diagnostic rate of symptoms related to ASD in this study was 34 %, higher than that observed by Ricotti et al. [19] at 21 % and Banihani et al. [20] at 15 %, and it was 100 % among patients in the Dp140-/Dp71- group. Other studies that sought to evaluate the correlation between ASD diagnosis and the underlying genotype found trends toward higher prevalences among patients with impairment of cerebral isoforms, but without statistical significance [19,21]. Darmahkasih et al. [22] a retrospective study involving 700 patients affected by DMD, found a diagnostic rate of ASD of 14.8 % in the group with impairment of Dp71 and only 6.4 % in the group with predicted normal cerebral isoforms, although statistical significance was not demonstrated. The present study adds evidence that Dp71 impairment is important for understanding the unclear relationship between *DMD* and ASD. Doorenweerd et al. [11] demonstrated strong co-expression between cerebral isoforms of dystrophin and genes implicated in ASD and intellectual disability (ID). Genes co-expressed with Dp71 were mainly involved in receptor-receptor interaction, as well as vascular development. Additionally, fibroblasts from Dp71-deficient mice showed reduced metabolic activity and altered migration and proliferation rates, indicating a role for this isoform in these neuronal processes [23].

The authors found a prevalence of 35.5 % of symptoms of OCD among participants, a result that is compatible with the higher prevalences of obsessive-compulsive behaviors in DMD patients compared to other children in the same age range described in the literature [24]. Pascual-Morena et al. [8] estimated an overall OCD prevalence of 12 % among DMD patients in a recent meta-analysis, compared to 1.23 % in the general population. Few studies have assessed the association between OCD manifestation and patient genotype [22,25]. Darmahkasih et al. [22] found similar prevalences among the isoform groups studied (Dp427 vs. Dp260, 140 and 116 vs. Dp71), ranging from 18 % to 25.7 %, with the lowest prevalence in the group with Dp71 impairment. Lambert et al. [25] did not observe any differences in OCD prevalence among the genotypic subgroups. In the only meta-analysis on the subject, the studied genotypes were not associated with a higher prevalence of OCD [14]. Patients with OCD exhibit significant changes in the volume and activity of the amygdala, a critical area for processing behaviors such as fear, anxiety, and reward primarily through glutamatergic pathways [26]. It has already been demonstrated that DMD expression in the adult human brain is high in the hippocampus and amygdala[11] Therefore, the loss of function of cerebral dystrophin could explain the manifestation of obsessive-compulsive symptoms in DMD patients, either through glutamatergic receptor dysfunction or amygdala neuronal architecture disorganization. The present study advances the understanding of the genesis of OCD in dystrophinopathies by finding an association between a higher prevalence of this disorder and the involvement of cerebral isoforms. Only one patient diagnosed with OCD belonged to the group with predicted intact cerebral isoforms. Further studies of larger sample sizes and diverse populations are needed to confirm the present findings.

The most common neurodevelopmental disorder among DMD patients appears to be ADHD, present in more than half of the present cohort. Pane et al. [27] found a 36.9 % positivity rate in a prospective study using DSM-IV criteria, a number similar to that found by Darmakahsih et al. [22] at 31 %. The association of this disorder with genotype is not a consensus. Ricotti et al. [19] reported an association between genotype and hyperactivity but not inattention, which was common in all groups evaluated, while Pane et al. [27], in a group of 103 patients, found a difference in the prevalence of ADHD among the genotypic subgroups of *DMD*, mainly for patients with mutations downstream of exon 63 and those with mutations in exons 45–55. Thangarajh et al. [28] in 193 steroid-naive boys found no difference between the upstream or downstream *DMD* exon 45 groups.

The landscape of dystrophinopathies has changed considerably over the past two decades, with increased life expectancy making cognitive/behavioral manifestations key contributors to the overall disease burden [29]. This trend is expected to intensify, as novel disease-modifying therapies are predominantly targeted at muscle tissue or fail to cross the blood-brain barrier. Consequently, addressing non-motor symptoms will become a critical unmet need in the future, with understanding these dimensions being essential for the development of therapeutics aimed at central nervous system manifestations.

The multicenter study evaluated patients with DMD and BMD from different social, ethnic, and genetic strata, using validated instruments applied prospectively by a group of trained physicians and psychologists to minimize internal inconsistencies. Most participants had not received previously comprehensive evaluations for neurodevelopmental disorders. Among the main study limitations is the overrepresentation of participants with DMD, with the data's validity being better suited to DMD patients. Due to the limited sample size, the authors did not perform analyses exclusively for the DMD subgroup or the BMD subgroup, which may introduce bias related to the inclusion of BMD cases. However, since these entities are contemporarily considered spectra of the same condition and the location of variants can influence non-motor phenotypes across the entire spectrum, the authors believe that this analysis holds greater external validity for its findings in patients within the dystrophinopathy group. Another significant limitation is that 39 out of 50 participants were using corticosteroids, the standard treatment for DMD, which has considerable neuropsychiatric adverse effects such as cognitive impairments, behavioral changes, and long-term psychiatric disorders [30]. The use of corticosteroids among the isoforms did not differ in regimen or dose, and the only theoretical impact of the treatment on the results could be related to the slightly higher prevalence of different disorders in the entire sample compared to the literature. No statistical corrections were made for the use of corticosteroids due to the small sample size of steroid-naïve participants, but studies that made such comparisons [27,28] did not find a clear relationship between steroid regimen and ADHD, and in many patients, pre-existing hyperactivity symptoms were the primary contraindication for use. Another limitation is that the scales used are more geared towards grading severity rather than diagnosing the neurodevelopmental conditions being evaluated. However, as there are thresholds suggesting symptoms consistent with such diagnoses and since assessments could only be done remotely due to the context of the COVID-19 pandemic, this was the feasible strategy to address the research question. It is worth mentioning that the results indicate a gradient where the more cerebral isoforms are affected, the higher the CARS score, supporting the findings also found in the categorical analysis.

In conclusion, the prevalence of symptoms compatible with ASD and OCD among patients with dystrophinopathies is related to the position of the causal variant in *DMD* and the consequent involvement of cerebral isoforms, indicating an important genotype-phenotype correlation. The diagnosis of a patient with a genotype that affects these isoforms indicates the need for neuropsychological assessment and multidisciplinary follow-up.

## Authors’ contributions

FMS, GMG and JAMS, contributed to the conception and design of the study. FMS, BFR, DLR, and ACBF contributed with the acquisition and analysis of data. FMS and JAMS contributed to drafting the article. MMB contributed critically to revising the manuscript for important intellectual content. All authors have approved the final version of this article.

## Funding

The study was funded by Financiamento e Incentivo à Pesquisa do Hospital de Clínicas de Porto Alegre (FIPE-HCPA) (Grant Number: 2019–0384), and Coordenação de Aperfeiçoamento de Pessoal de Nível Superior (CAPES) (Grant Number: PROEX: 0730/2020). Saute JA was supported by Conselho Nacional de Desenvolvimento Científico e Tecnológico (CNPq), Rosa BF by Fundação de Amparo à Pesquisa do Estado do Rio Grande do Sul (FAPERGS) and HCPA, and Rocha DL by Coordenação de Aperfeiçoamento de Pessoal de Nível Superior (CAPES).

## Data availability

Data not provided in the article because of space limitations may be shared (anonymized) at the request of any qualified investigator for purposes of replicating procedures and results.

## Conflicts of interest

The authors declare no conflicts of interest.

## Appendix Supplementary materials

Image, application 1

## Footnotes

Supplementary material associated with this article can be found in the online version at doi:10.1016/j.jped.2025.01.014.
