# Supplementary material for: Genotype-phenotype correlation of neurodevelopmental disorders in patients with dystrophinopathies
Source: J Pediatr (Rio J). 2025 Jun 6;101(4):536–43. doi: 10.1016/j.jped.2025.01.014 (PMC12276596; doi:10.1016/j.jped.2025.01.014)
Supplement: Supplementary file 1 [file mmc1.docx]

**JPED-D-24-00192_Supplementary Material**

**
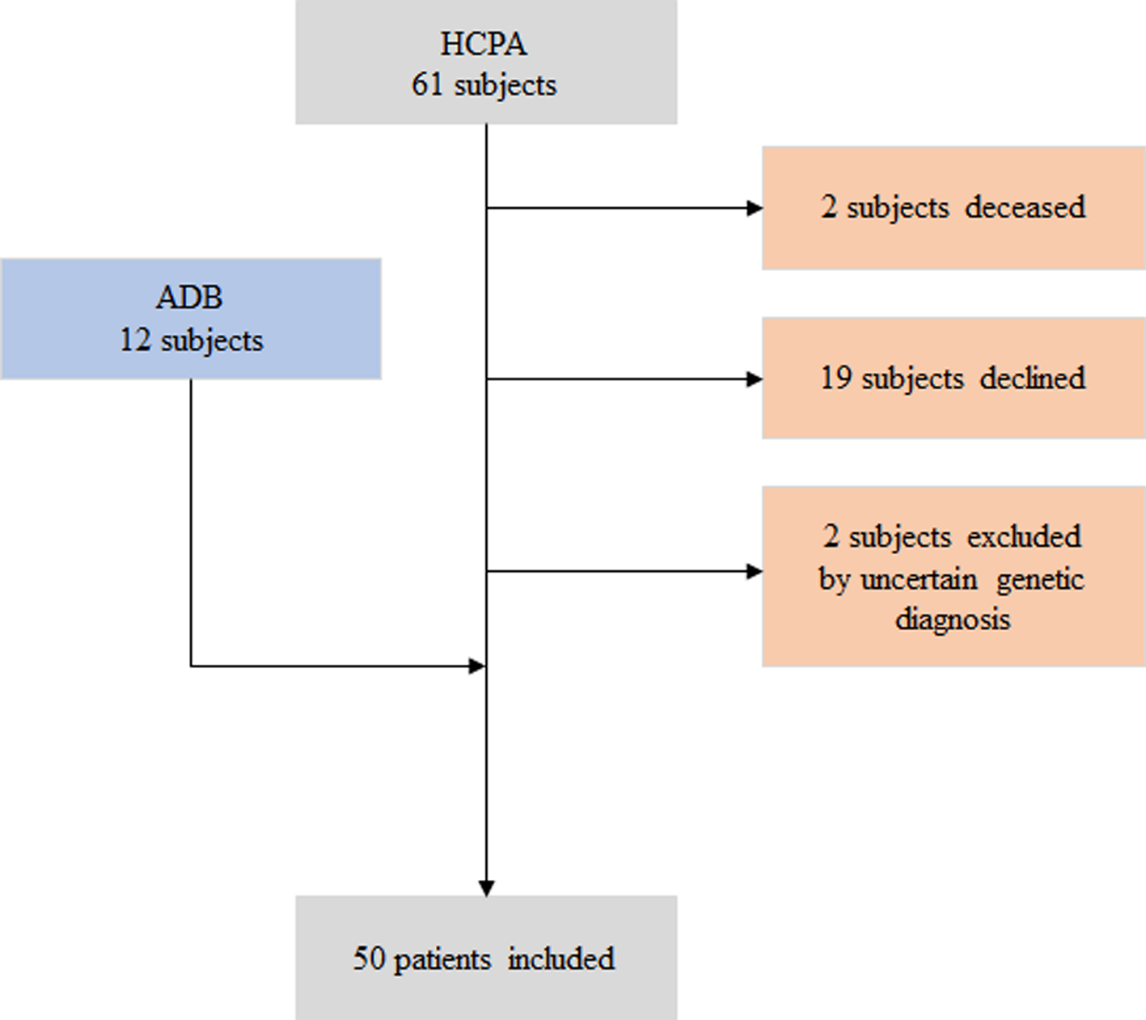
**

**Supplementary Figure 1 Recruitment Flowchart.** ADB, Aliança Distrofia Brasil; HCPA, Hospital de Clínicas de Porto Alegre.

**
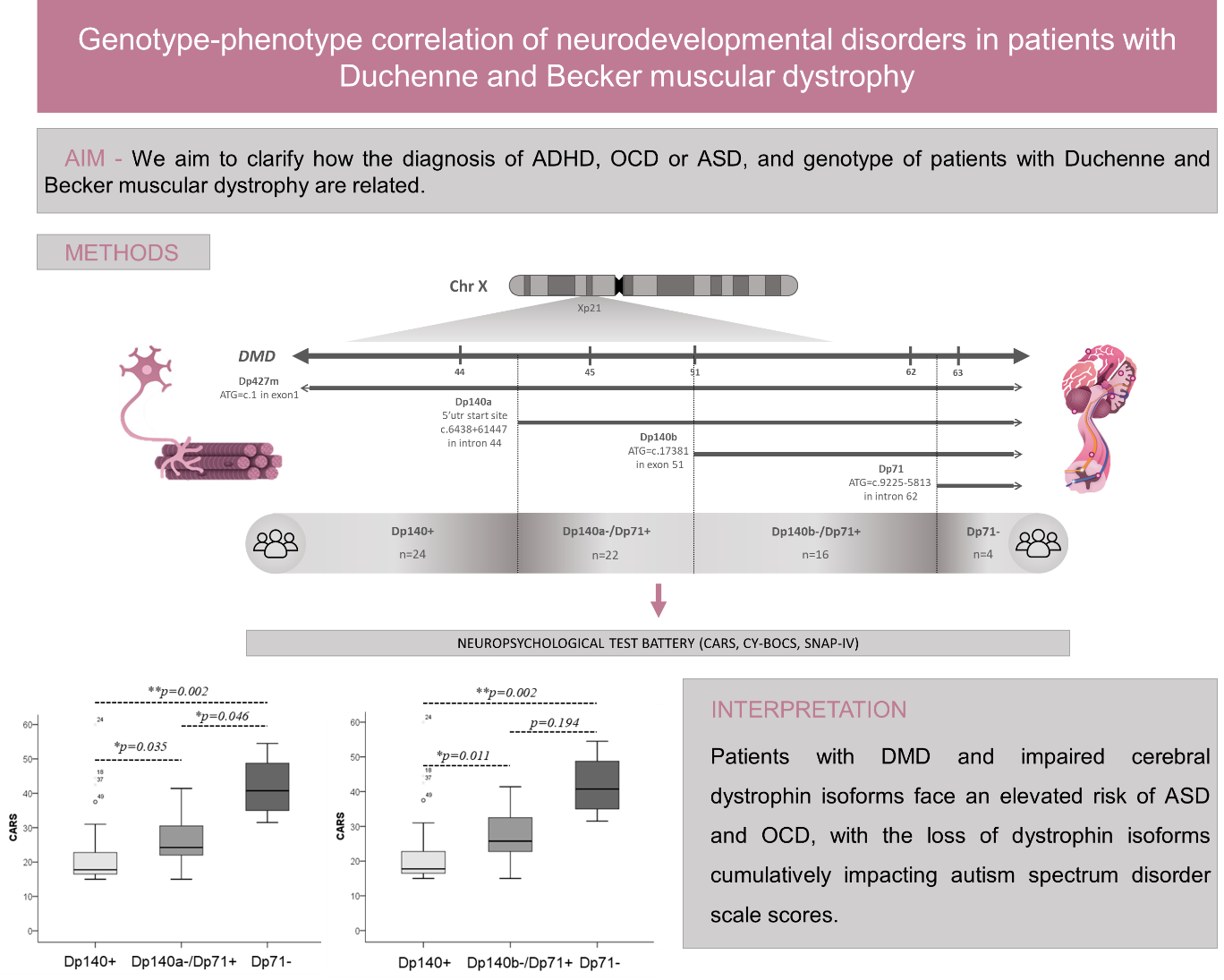
**

**Graphical Abstract**

ADHD, Attention-Deficit/Hyperactivity Disorder; ASD, Autism Spectrum Disorder; CARS, Childhood Autism Rating Scale; CY-BOCS, Yale-Brown Obsessive Compulsive Scale for Children; DMD, Duchenne Muscular Dystrophy; OCD, Obsessive-Compulsive Disorder (OCD).
